# Towards A Physics-based Model for Steerable Eversion Growing Robots

**DOI:** 10.1109/LRA.2023.3234823

**Published:** 2023-02

**Authors:** Zicong Wu, Mikel De Iturrate Reyzabal, S.M.Hadi Sadati, Hongbin Liu, Sebastien Ourselin, Daniel Leff, Robert K. Katzschmann, Kawal Rhode, Christos Bergeles

**Affiliations:** School of Biomedical Engineering & Imaging Sciences, King’s College London, London, UK; Faculty of Medicine, Department of Surgery & Cancer, and the Hamlyn Centre for Robotic Surgery, Imperial College London, London, UK; Soft Robotics Lab, ETH Zurich, Switzerland; School of Biomedical Engineering & Imaging Sciences, King’s College London, London, UK

## Abstract

Soft robots that grow through eversion/apical extension can effectively navigate fragile environments such as ducts and vessels inside the human body. This paper presents the physics-based model of a miniature steerable eversion growing robot. We demonstrate the robot’s growing, steering, stiffening and interaction capabilities. The interaction between two robot-internal components is explored, i.e., a steerable catheter for robot tip orientation, and a growing sheath for robot elongation/retraction. The behavior of the growing robot under different inner pressures and external tip forces is investigated. Simulations are carried out within the SOFA framework. Extensive experimentation with a physical robot setup demonstrates agreement with the simulations. The comparison demonstrates a mean absolute error of 10 - 20% between simulation and experimental results for curvature values, including catheter-only experiments, sheath-only experiments and full system experiments. To our knowledge, this is the first work to explore physics-based modelling of a tendon-driven steerable eversion growing robot. While our work is motivated by early breast cancer detection through mammary duct inspection and uses our MAMMOBOT robot prototype, our approach is general and relevant to similar growing robots.

## I Introduction

SOFT eversion growing, or vine robots, change their length by extending their tip usually through deposition and curing of a thermo-active material similar to the 3D-printing process [1], or eversion rolling of an in-extensible but highly bendable tube via hydraulic/pneumatic pressure [2]. In both designs, the robot navigates via “adding” material to its tip.

Our manuscript focuses on robots that grow via tip eversion, which avoids the translational motion between the robot body and its surroundings. This is key for navigation in confined or narrow spaces [2], such as archaeological sites [3], disaster sites [4], and anatomical lumens [5], [6]. Passive steering may be achieved thanks to preformed pinched locations on the growing element [2], [6]. Active steering is possible by changing the structural stiffness of the soft growing sheath. Tendons, pneumatic/hydraulic pressurisation, and smart artificial muscles can be utilised [7]. Various application-specific units can be integrated at the tip of growing robots, from sensing units [3] to surgical instruments [5].

Modelling a growing robot’s mechanics underpins its control for navigation and exploration. A kinematic model and obstacle interaction model have been established to facilitate robot path planning in [8]. David *et al*. proposed a comprehensive mathematical model to describe a robot’s behavior during environmental interaction by incorporating transverse and axial buckling modes [9]. A dynamics simulator based on pseudo-rigid-body kinematics with contact constraints is proposed to investigate behavior of soft growing robots in cluttered environments, but neglecting the robot continuous length change and the sheath translation dynamics [10]. In an earlier work, we presented the first dynamic model of such robots capturing the coaxial translation and continuous growth of an everting sheath based on a Reduced Order Model via spline shape fitting kinematics [5]. The interaction between robot and environment, the stiffening effect of everting sheath internal pressure, and physics-based exact dynamic modelling of such a system, however, are yet underexplored.

A physics-based model can provide better insight into the real physics of a soft robot by providing a geometrically exact model of the system, compared to simplified theoretical models based on Constant Curvature, piecewise kinematics, or modal solutions [11]. Finite Element Modelling (FEM) has been a popular tool for physics-based modelling and simulation of soft robots in recent years with meshes based on 3D volumetric, 2D planar, or 1D linear element [12]. In [13], the FEM method is used with quadratic programming to control the locomotion and manipulation of a soft robot, taking into account the friction force between the robot and multiple objects, as well as trajectory optimization [14]. Several controllers, also based on real-time FEM, have been proposed to control soft robots [15], [16]. FEM also enables acquisition of training data to learn the kinematic model of a soft robot using artificial neural networks [17].

Simulation Open Framework Architecture (SOFA) is a cross-disciplinary open-source library that can simulate interactions within physical environments [18]. It has gained popularity in design optimization, modelling, and simulation of soft robots and actuators [19]–[22]. However, despite the potential of SOFA in modelling soft robots, it is not trivial and there has been no instance of a physics-based model for an eversion growing robot in the literature.

**Fig. 1:**
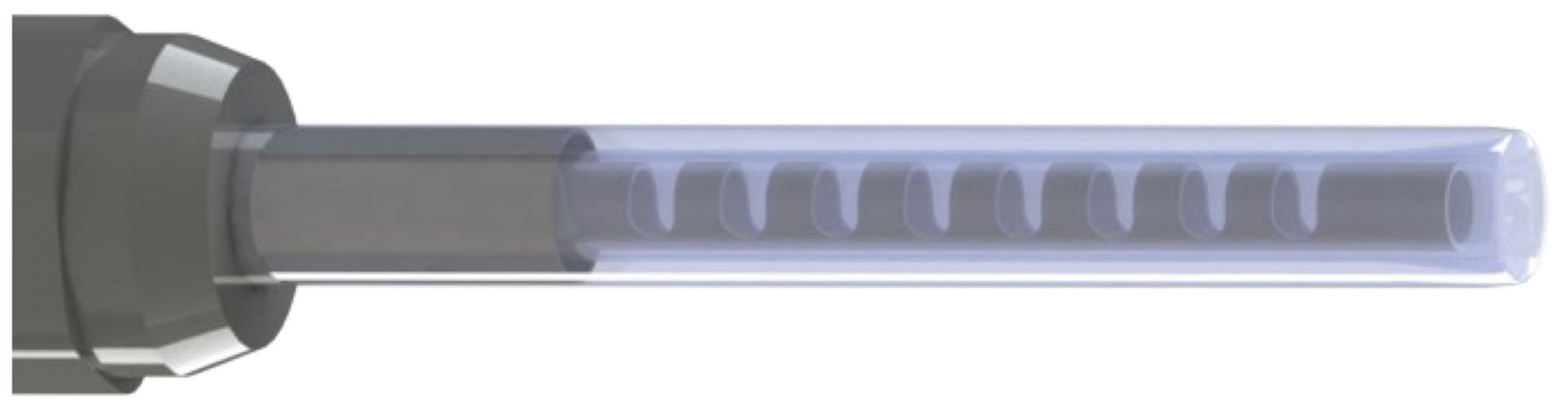
The MAMMOBOT tip consists of a soft growing sheath covering a steerable catheter for navigation within the mammary duct branching lumen.

In our paper, a steerable soft growing robot, shown in Fig. 1, was built based on our previous work [5]. The robot is envisioned for early breast cancer detection through mammary duct inspection, and is termed MAMMOBOT (see summary in Sec. II). The digital twin of its key elements, the eversion growing element and steerable catheter, was established in SOFA, as the first instance of a physics-based model for an eversion growing robot. Physics-based modelling of growing, steering, and interaction with the environment using SOFA is detailed in Sec. III. Finally, the simulation results and comparison with benchtop experiments are provided in Sec. IV. The manuscript concludes in Sec. V.

To the best of our knowledge, this manuscript is the first to tackle the physics-based modelling and experimental validation of eversion growing robot behavior under complex interaction with the environment, including the effect of internal pressure on the growing and stiffening features of such systems. Our results highlighted the advantages and shortcomings of a physics-based model for such complex systems. Our SOFA scenes are available as open source plugin^1^.

## II Robot Setup Structure

**Fig. 2:**
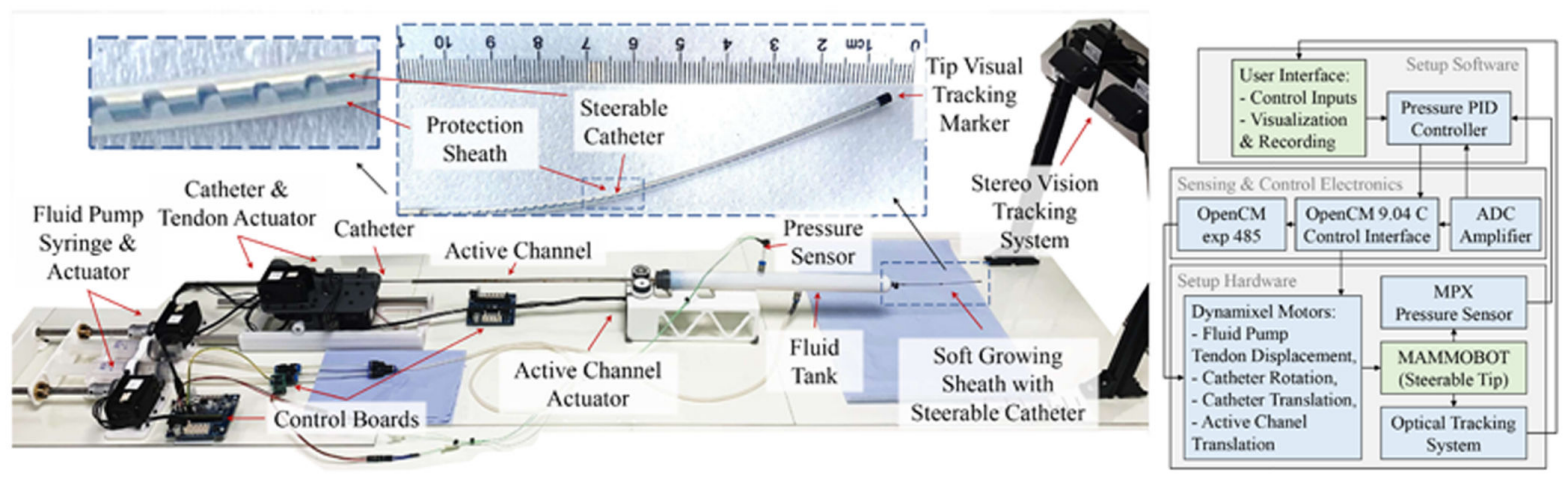
(**Left**) MAMMOBOT’s new design and its key elements. (**Right**) System Control Diagram.

MAMMOBOT, shown in Fig. 2, was first presented in [5] and comprises three subsystems: (1) a base platform enabling insertion, rotation, and deflection of a steerable catheter using a tendon, (2) a syringe pump system to regulate the hydraulic pressure for growing and retraction of a soft growing sheath, and (3) a pressurised saline-filled tank system integrating the soft growing element and steerable catheter. The architecture maintains an inner lumen to deliver tools like a miniature endoscope, biopsy and imaging probe to the intervention site.

In support of the experimental evaluation of this manuscript, we improved on the original MAMMOBOT in a number of ways. The original Nema series motors (Eco-Worthy, China) were replaced by advanced Dynamixel AX64 smart actuation units (Robotis Inc., USA). These motors encompass encoder, reduction gear and torque feedback. The saline-filled tank was redesigned to address leaking under the high hydraulic pressures required to achieve robot growth. It was fabricated using CONNEX3 OBJET500 printer (Stratasys Inc., USA) with Durus polypropylene material. Push-fit components were employed to facilitate connection with a pressure sensor and fluid management tubing. The soft growing element was fabricated per our established approach [5], using LDPE sheets (Polybags, UK) with a thickness of 35 μm. The length of the soft growing element is 28 cm, which can reach the theoretical maximum growth range of 14 cm (accounting for the fact that half the length must remain everted). To optimize sealing at the back end, rubber O-rings, nitrile rubber V-shape rotary shaft seal, and customized elastic gasket using OOMOO™ 30 silicon rubber (SMOOTH-ON Inc., USA) were utilized. When not specified, the parts of the platform were fabricated using a Formlabs 3B SLA printer (Formlabs Inc., US), and an Delta WASP 2040 INDUSTRIAL X FDM printer (WASP S.r.l., Italy) with resin and PLA material, respectively.

## III Physics-Based Model Implementation in SOFA

To implement a physics-based model of a robot in SOFA, a simulation environment (scene) should be created to match the robot physical counterpart. SOFA enables users to use a variety of modules to define different elements of a robot as separate nodes and their interactions via different collision models. Although it is possible to implement user-specific elements such as specific kinematic assumptions or material laws, in this paper, we focused on exploiting the available SOFA tools to model an eversion growing robot. This enabled us to estimate the capability of the available SOFA toolset for modeling the complex structure of a growing robot, to reflect on its limitations, and to provide suggestions for the necessary developments to fully capture the mechanics of a steerable eversion growing robot. The details of the implementation of MAMMOBOT are provided in Algorithm 1.

**Algorithm 1 The Implemented Scene in SOFA Framework**

**Node** ’root’

        *SimulationParameters* ← dt, gravity, CollisionPipeline

        **Node** ’Sheath’:

                *SheathLinearSolvers*

                *MechanicalObject* ← sheath-bounding-box

                *MeshSpringForceField* ← Physical parameters

                *addConstraints* ← Base indices

                **Node** ’Sheath Surface’: ← *PressureField*

                **Node** ’Sheath Collision’: ← *SurfaceSpheres*

                **Node** ’Guide Collision’: ← *CenterLineSpheres*

        **Node** ’Catheter’:

                *CatheterLinearSolvers*

                *MechanicalObject* ← catheter-bounding-box

                *MeshSpringForceField* ← Physical parameters

                *addConstraints* ← Base indices

                **Node** ’Tendon’: ← *MeshSpringForceField*

                **Node** ’Catheter Collision’: ← *SurfaceSpheres*

        **PythonController** ’SheathCatheterController’

**Fig. 3:**
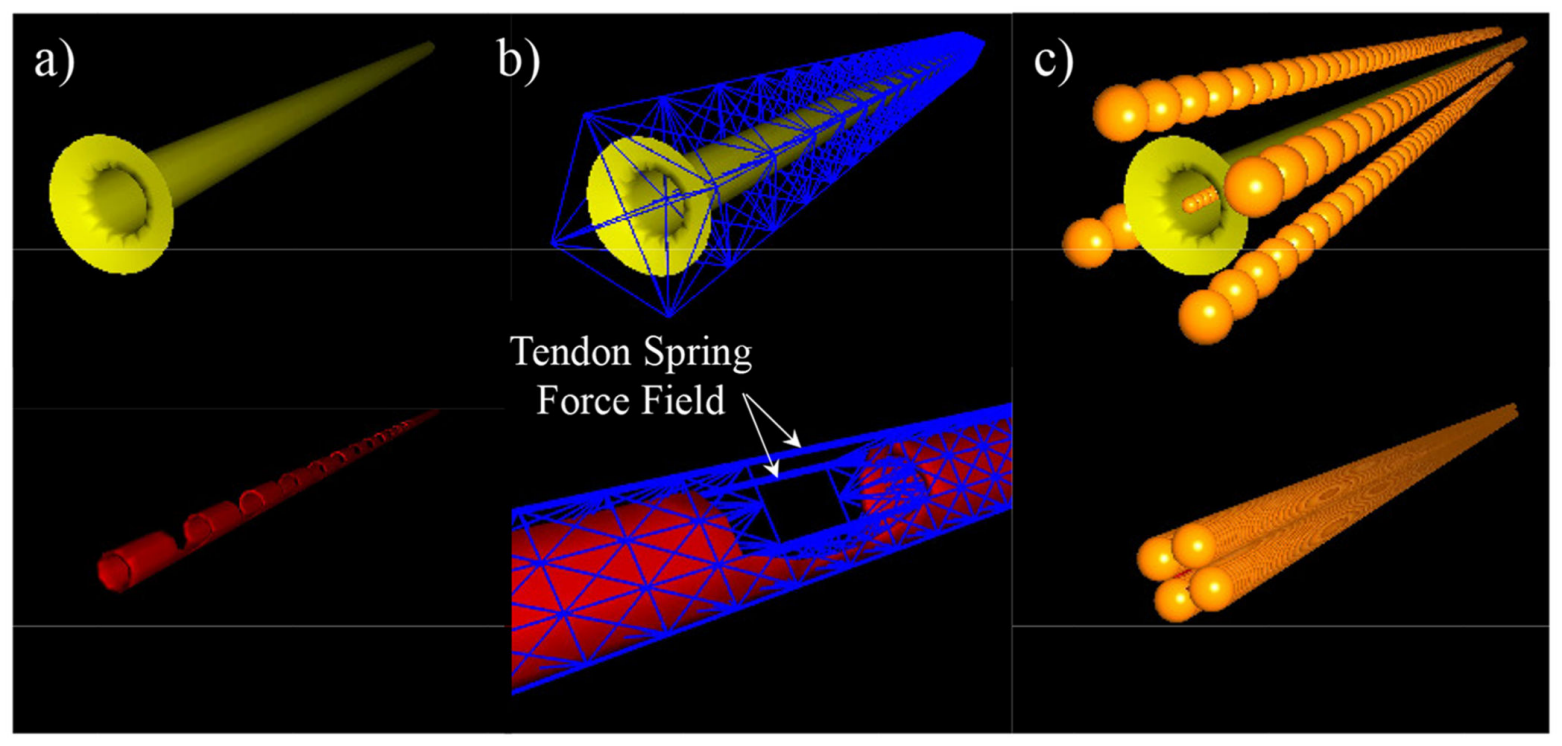
Multi-model representation of: (**Top**) sheath, and (**Bottom**) catheter. (a) Visual Mesh, (b) Physical Sparse Grid, and (c) Spherical Collision Model.

Every element in the simulation is represented using a sub-model that contains information about the interaction with other elements and environment. Such a multi-model representation enabled us to differentiate different physical, visual, and collision models inside our simulation (see Fig. 3) and to present the first physics-based dynamic model for the eversion growing of a soft robot.

The simulation parameters were set via manual measurements for the dimensions and weight. The stiffness and damping coefficients are set based on the material Young’s modulus and then fine-tuned to match the experimental results with a stable damped behavior (see Table I).

### A Sparse Grid Topology & Mechanical Object

The OBJ modelling files of the simulation targets were created based on the CAD drawing file of MAMMOBOT and imported into SOFA. Meshing of each object was then implemented; its bounding box was discretized in the simulation space to effectively compute its geometric information. In our implementation, discretization was performed using a *SparseGridTopology* to reduce the edges required for each mechanical object and therefore speed up computation. A 3D volumetric or 2D planar mesh could not replicate the incompressible behavior of a growing sheath which is necessary to successfully capture the sheath eversion growing physics. This is because of the volume or area preservation constraints commonly associated with those meshing techniques.

### B Simulation Principles

#### 1) Animation Loop Time Step & Gravity

A Default Animation Loop was used for the simulation that defines the order of simulation steps within the scene. The simulation time step, dt, was optimized to reduce the computation time while achieving a stable eversion growth as 0.01 s. Gravity was ignored when only the sheath eversion was evaluated. Otherwise, gravitational acceleration was set to 9.81*e*3 mm/s^2^.

#### 2) Boundary Conditions

The bases of the sheath and catheter were fixed to their initial position in space, mimicking the physical setup and with them being the robot’s exit point from the pressurised chamber.

#### 3) Collision Pipeline

A Default Pipeline was defined to perform collision detection and handling. The *DefaultContactResponse* module was used that models the contacts using springs with assigned stiffness, defined on each of the object nodes, for every colliding element. A drawback of such a collision model is that it cannot effectively model the friction between the contacting elements. Alternative collision models that could successfully capture a frictional contact between the elements hindered the simulation computational performance significantly and did not result in a stable scene.

We could only achieve a penetration-free and stable simulation of the eversion growing based on a *SphereCollisionModel*. The sphere radius of the collision models was selected in a way that the sheath and catheter visual models, which were based on 2D planar meshes of these geometries, did not penetrate upon their interaction while resulting in a stable simulation of the system growing, bending, and rotation.

#### 4) Linear Systems and Solvers

The simulation of continuum mechanics in computational graphics always involves deriving the solutions to a linear system. In our implementation, this linear system was handled using SOFA’s *EulerImplicit-Solver* with *rayleighStiffness* and *rayleighMass* set as 0.001 for the growing sheath and 0.1 for the catheter to achieve a damped system behavior. The *CGLinearSolver* was used to iteratively determine the approximate solution without prior knowledge of the system, following the *conjugate gradient* method. The tolerance and threshold of *CGLinearSolver* were both set to 10^−5^, with an iteration number of 30, for a balance between the simulation numerical performance and stability.

### C System Elements (Nodes)

#### 1) Soft Eversion Growing Sheath

The soft growing sheath was modelled as a thin-walled structure with a cone shape to direct the pressure field towards its tip. A spring force field was assigned to the discretized bounding box using *MeshSpringForceField*, with stiffness and damping value set as 3 × 10^8^ and 40, respectively. One end of sheath was fixed in place to its initial position using *FixedConstraint*. This ensured that the growing end of the sheath moved at a constant controllable velocity. A controlled relative pressure with an initial amplitude of 50 Pa was applied along the normal directions of a 2D mesh of the sheath geometry. This *SurfacePressureForceField* compresses the everting inner part of the sheath to the steerable catheter while expanding its everted stationary outer part. A *SphereCollisionModel* was implemented with the contact stiffness of 100 and the restitution coefficient was set as 0.1. As the defined collision pipeline ignores contact friction, it was set to 0.

#### 2) Tendon-Driven Steerable Catheter

The steerable catheter was modelled as a 1x180x5 grid using *SparseGrid-Topology*. A *MeshSpringForceField* was generated to describe the catheter mechanics with stiffness and damping values of 8 × 10 ^8^ and 40, respectively. The *SphereCollisionModel* was implemented as described for the soft growing sheath. To deflect the steerable catheter, a tendon was approximated by connecting nodes on the edge of the catheter grid mesh along its axial direction. Its *MeshSpringForceField* was generated with *lineStiffness* of 2 × 10^9^, and *lineDamping* of 40, respectively. The pulling and releasing effect was modelled by updating the resting position of the tendon edges. One of the ends was fixed to its initial position similar to the case for the sheath. The catheter was grown and rotated using a constant velocity. the catheter translation velocity was set to half of the sheath’s base velocity to account for the sheath eversion.

### D SofaPython3 Controller

**Fig. 4:**
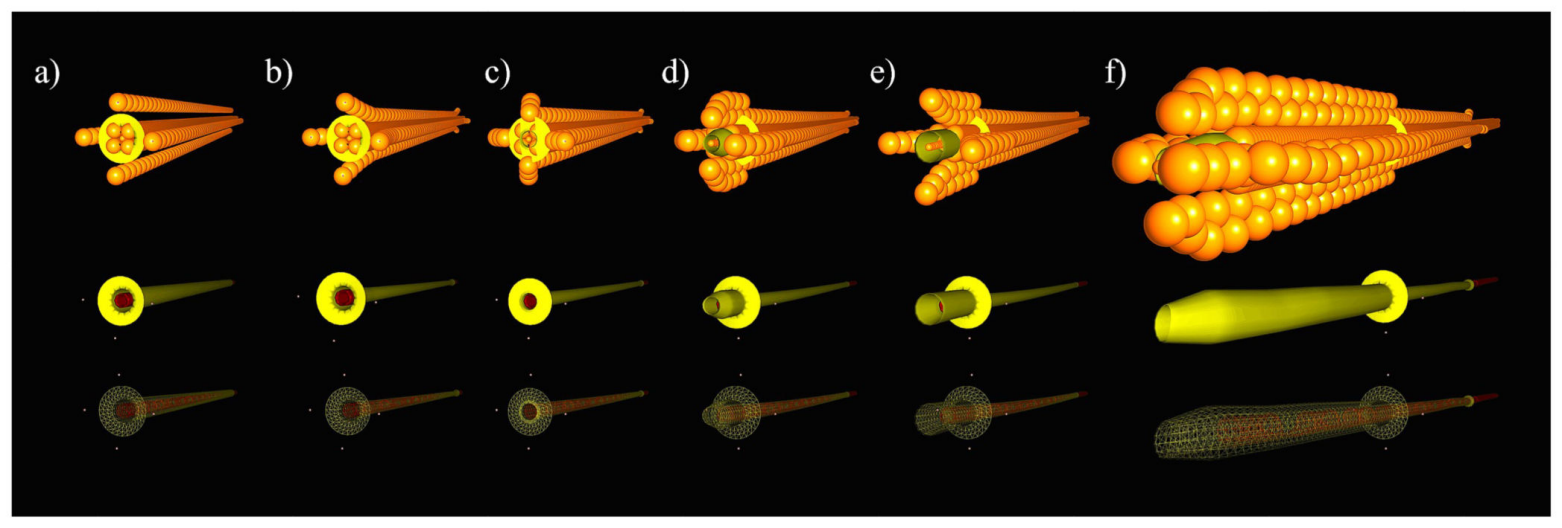
Dynamic simulation instances of MAMMOBOT during eversion growing (a) Initial system shape, (b) sheath shrinkage on the catheter after inducing pressure, (c) start of the pressure-induced eversion growing, (d)-(f) growing steps until reaching a predefined length. (**Top**) the spherical collision models for the sheath and catheter, (**Middle & Bottom**) shaded & wire frame rendering of the system during the eversion growing motion.

**Fig. 5:**
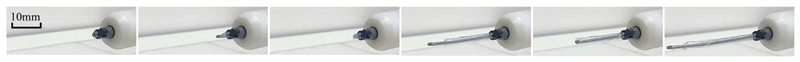
Eversion growing of MAMMOBOT, consisting of repeated insertion and retraction of the inner catheter to facilitate the eversion of the pressurized sheath (duty cycle approach). The first two left instances correlate with Fig. 4.a-e.

A control unit was implemented to interact with the scene, defined by the *AnimationLoop*. This enabled us to script SOFA simulations based on our experimental scenarios. Fig. 4 demonstrates the growing sequence in the simulated scene. The growth of the real system is presented in Fig. 5. Fig. 4.a-e highlights the first few simulation steps when the soft sheath eversion was initiated. During these steps, the visible change in the real system, i.e. the first instance in Fig. 5, is minimal.

## IV Experimental Results & Discussions

A series of experiments were carried out on both the physical robot and its digital twin in SOFA. The comparisons progressively increase the complexity of the experiments, starting from studies to tune the simulation parameters and leading to evaluation of the complete system behavior under different internal pressure and external loading cases. Error analysis are conducted based on the curvature values to avoid infinite values for the curve radius close to a straight configuration. Summary of the error analysis is presented in Table II.

### A Lateral Bending Experiments of Steerable Catheter

In this set of experiments, forces with different amplitudes and orientations were loaded at the catheter’s tip to determine the relationship between the catheter’s curvature, loading, and length-change of tendon. The catheter itself was slightly deflected due to assembly misalignments and manufacturing errors, and contains a small pre-curvature of 2.2×10^−3^ mm^−1^. This pre-curvature was subtracted from curvature calculations.

#### 1) Catheter curvature vs loading

**Fig. 6:**
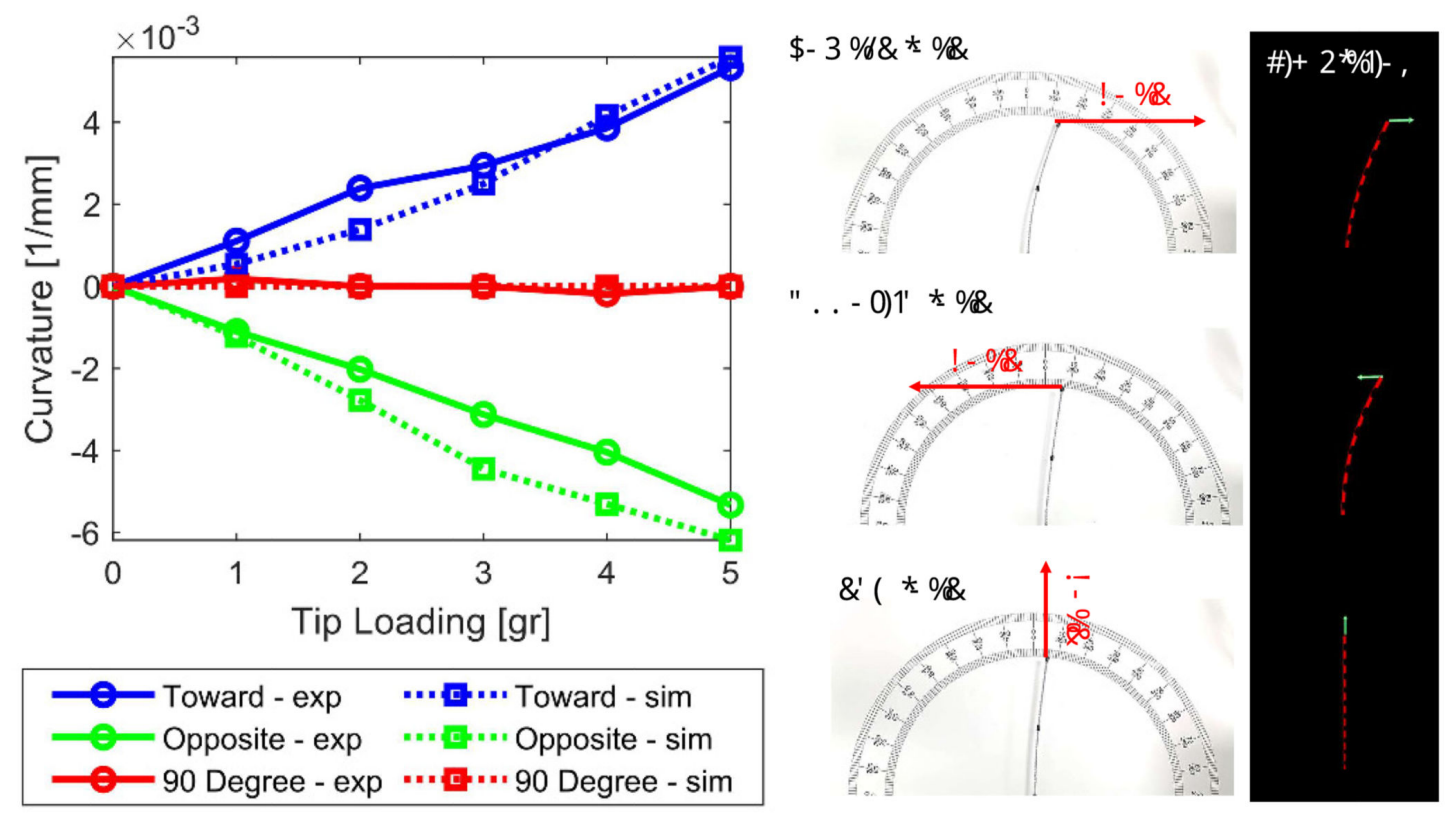
The steerable catheter’s curvature vs different loading values.

In the first experiment, the tendon was removed to consider only the stiffness of the catheter. Loading was applied to the tendon-free catheter to find out the relationship between the catheter’s curvature and tip loading. Results are shown in Fig. 6, wherein a solid line relates to the physical experiments and a dotted line to the outputs of the SOFA simulation.

The left column of Fig. 6 captures the relationship between the catheter’s curvature and loading force, when tip force is loaded along three different directions. The three directions correspond to: (**Toward - exp**) following the catheter’s deflection, (**Opposite - exp**) opposing the catheter’s deflection, and (**90 Degree - exp**) perpendicular to the cutting pattern on the steerable catheter. The middle column of Fig. 6 illustrates the physical setup, and the right column the simulation.

It can be observed that when the tip force is loaded opposite or towards the catheter’s deflection, the catheter’s curvature is (almost) linearly increasing with the loading force. When the tip force is loaded perpendicular to the deflection, there is no significant change in the catheter’s curvature. The MAE (Mean Absolute Error) between physical experiment and SOFA simulation is 0.43m^−1^, 0.72m^−1^, and 0.06 m^−1^ for toward, opposite, and perpendicular loading experiment, respectively. Considering the results from (**90 Degree - exp**) are all close to zero, the maximum error and percentage error are only calculated for the results from the other two loading directions. The overall MAE % is ≈ 25%.

#### 2) Catheter curvature vs tendon pull

In the second experiment, we investigated how tendon length change determines the catheter’s curvature. The tendon actuating the catheter was pulled up to a maximum of 3.3 mm.

**Fig. 7:**
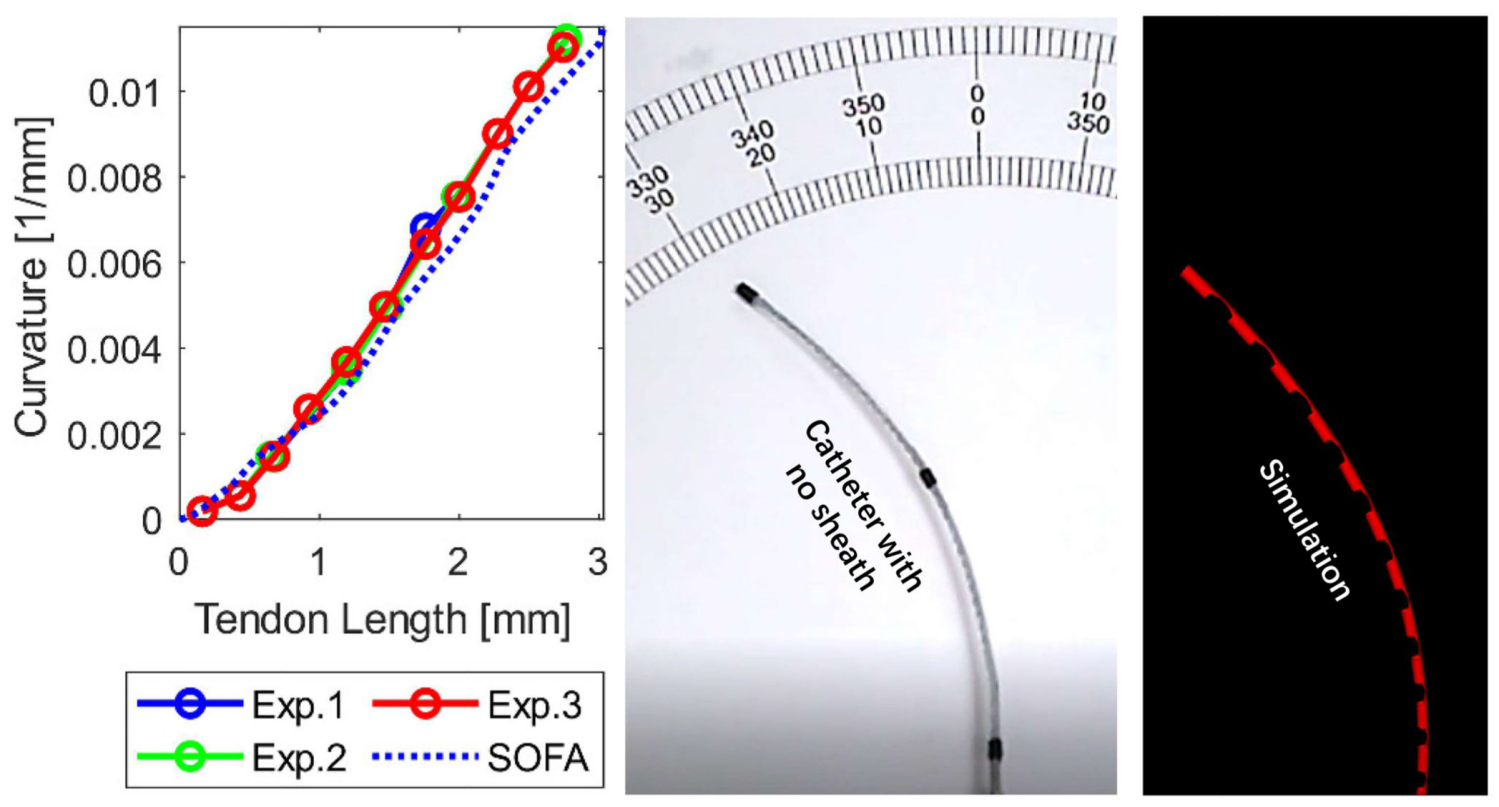
Steerable catheter (95 mm long) curvature vs tendon length change.

Fig. 7 captures the linear relationship between the catheter’s curvature and length change of the driving tendon. The MAE (%) is 0.55m^−1^ (20.78%). We note that the error increases as the tendon is pulled, but, overall, the slope corresponding to the physical experiment matches well the one corresponding to the simulations. The concordance of the comparisons in Fig. 6 and Fig. 7 suggest that the catheter’s digital twin parameters have been successfully tuned, therefore enabling further evaluation of the system’s components.

### B Growing Experiments of Sheath

**Fig. 8:**
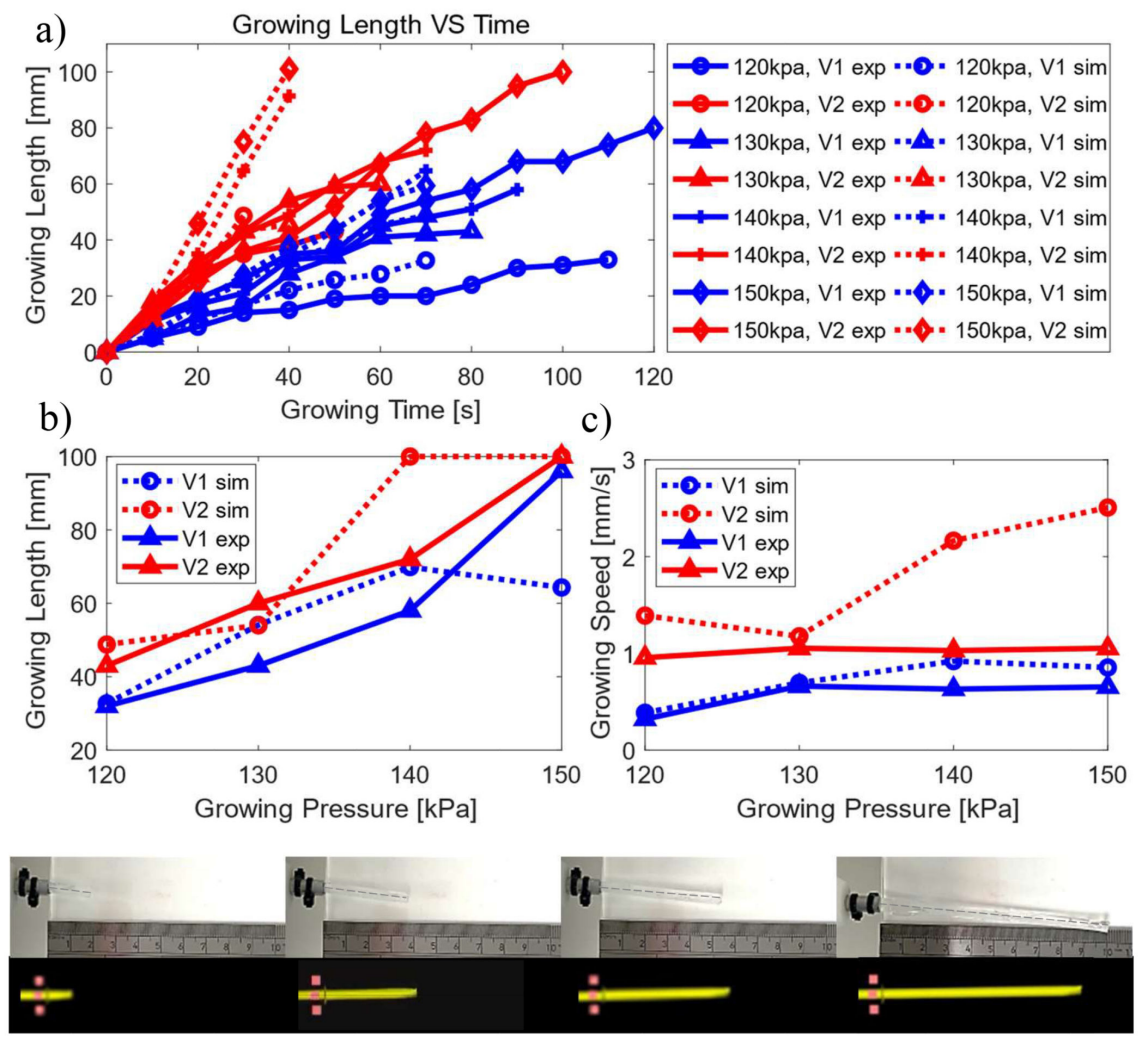
Sheath growing length and speed vs. active channel feeding speed (V1 = 2.9 mm/s, V2 = 5.8 mm/s) and inner pressure. (a) Growing length vs time; (b) Final grown length; (c) Average growing speed.

Growing experiments of a standalone sheath were carried out both experimentally and in SOFA to investigate the effect of pressure on the growing process. First, the pressure within the sheath was regulated and maintained at varying constant levels to actuate its growth. Pressures ranging from 120 kPa to 150 kPa were explored. Growing was not achieved for smaller pressures, while higher pressures occasionally led to sheath rupture. Second, as sheath growth is also affected by the feeding speed of the active channel, different speeds, namely *V*_1_ = 2.9 mm/s and *V*_2_ = 5.8 mm/s were evaluated within the growth experiments. Real-time growing length vs elapsed time, final growth length vs pressure, and average growing speed vs pressure for each experiment is shown in Fig. 8.

In Fig. 8(a), it can be seen that the slope of the plots increases both with the increasing pressure and the feeding speed of active channel. Moreover, pressure plays a more dominant role in growing, compared with feeding speed, which can be observed by comparing the results with controlled variable of either pressure or feeding speed.

Fig. 8(b) demonstrates that the final growing length increases with increased pressure. In addition, when feeding speed increased from *V*_1_ to *V*_2_, the final growing length also increased. This can be observed in both simulation and experimental results. It is worth noting, the final grown length tends to be convergent (does not grow further) in simulations with increasing growth pressure. However, experimental results demonstrate continuous growing until maximum length is reached. This might result from pressure fluctuation in the real system. Even though pressure is regulated, the inner pressure of the tank will always vary more than the simulated one. Rapid changes may lead to larger pressure-induced forces maintain growth. As summary, this experiment further supports the conclusions from Fig. 8(a) that pressure plays a more dominant role in growing, both for speed of growth and final length. The MAE (%) of final length is 11.89mm (17.97%).

Fig. 8(c) provides results comparing the average growing speed of the physical setup and the SOFA digital twin. Qualitatively, both setups show that sheath growth speed increases with feeding speed. In simulations, however, growing speed is not consistently affected by pressure levels. In more detail, for the lower insertion speed, *V*_1_, there is distinct concordance between the two experimental modalities. The MAE (%) in that case is 0.14 mm/s (25.59%). Experiment and simulation is also in qualitative agreement when a higher insertion speed, *V*_2_ is applied; MAE (%) is calculated at 0.78 mm/s (76.46%). Overall, the MAE (%) of average growing velocity is 0.46 mm/s (51.03%), which is mostly contributed by higher feeding speed of active channel.

To balance computing cost and simulation reliability, as discussed in Sec. III, a spring based contact model was used. These models are limited when modelling complex friction, as the case analysed in this experiment. The closest mutable variable to model these interactions is contact spring damping. However, this parameter can not be directly related to friction on the contacts and does not show a reliable trend on behaviour, as it has been well investigated during this research. Although the used contact model is not ideal, it balances computational complexity with fidelity.

### C Gravity Effect on Sheath

**Fig. 9:**
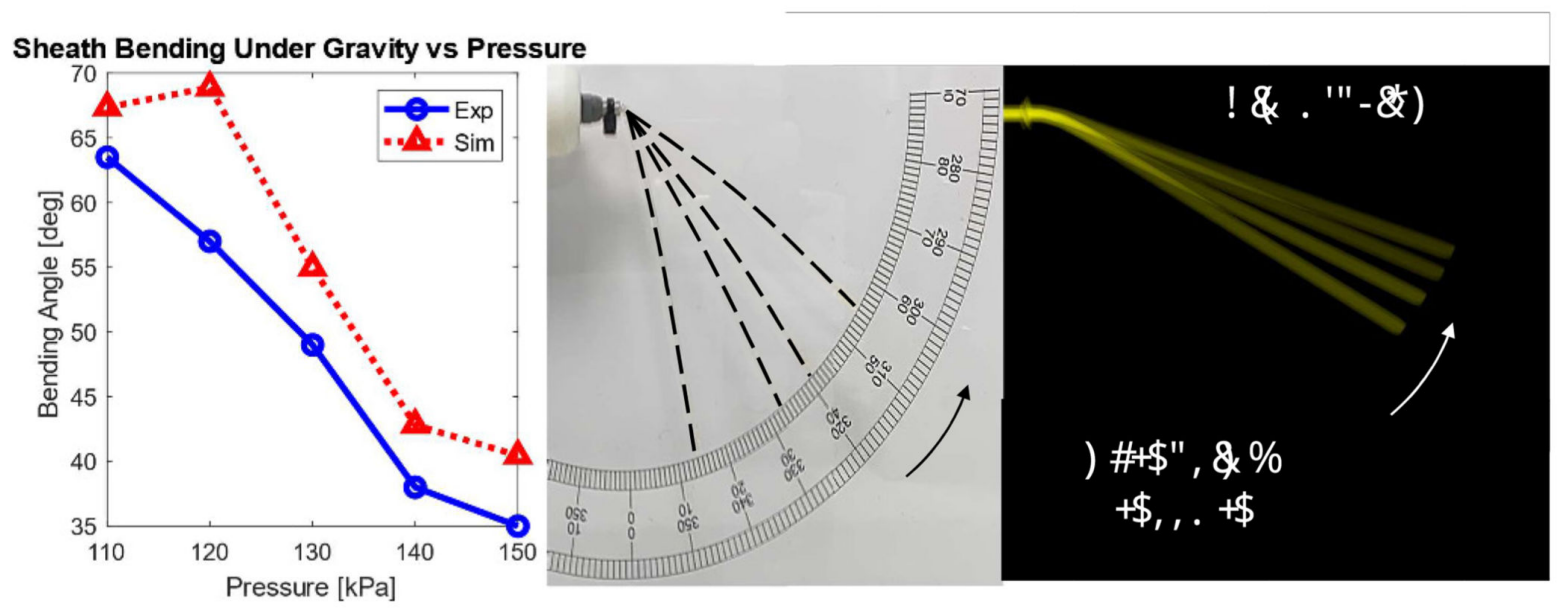
Bending angle of a suspended sheath (diameter 3 mm), under gravity.

It was experimentally observed that gravity, depending on the actuating pressure, induced vertical bending and affected robot growth, primarily due to the weight of the saline solution accumulated within the sheath. Therefore, this relationship was captured with relevant experiments. In both the physical setup and SOFA simulation, the robot was pressurised from 110 kPa to 150 kPa to stiffen it and counteract the effect of gravity. The results are shown in Fig. 9. The deflection angle of the growing sheath caused by gravity reduces with increasing inner pressure. This is anticipated, as increased pressure increases the sheath’s stiffness. The MAE (%) between comparative results is 6.39° (13.46%), which implies that the mass of the pressurised saline solution is correctly accounted for.

### D Lateral Stiffness Experiments

Steering of the eversion growing robot is affected by the inner pressure of the growing sheath. To investigate how pressure affects the full system performance, lateral stiffness experiments were carried out.

#### 1) Absence of Tip Load

**Fig. 10:**
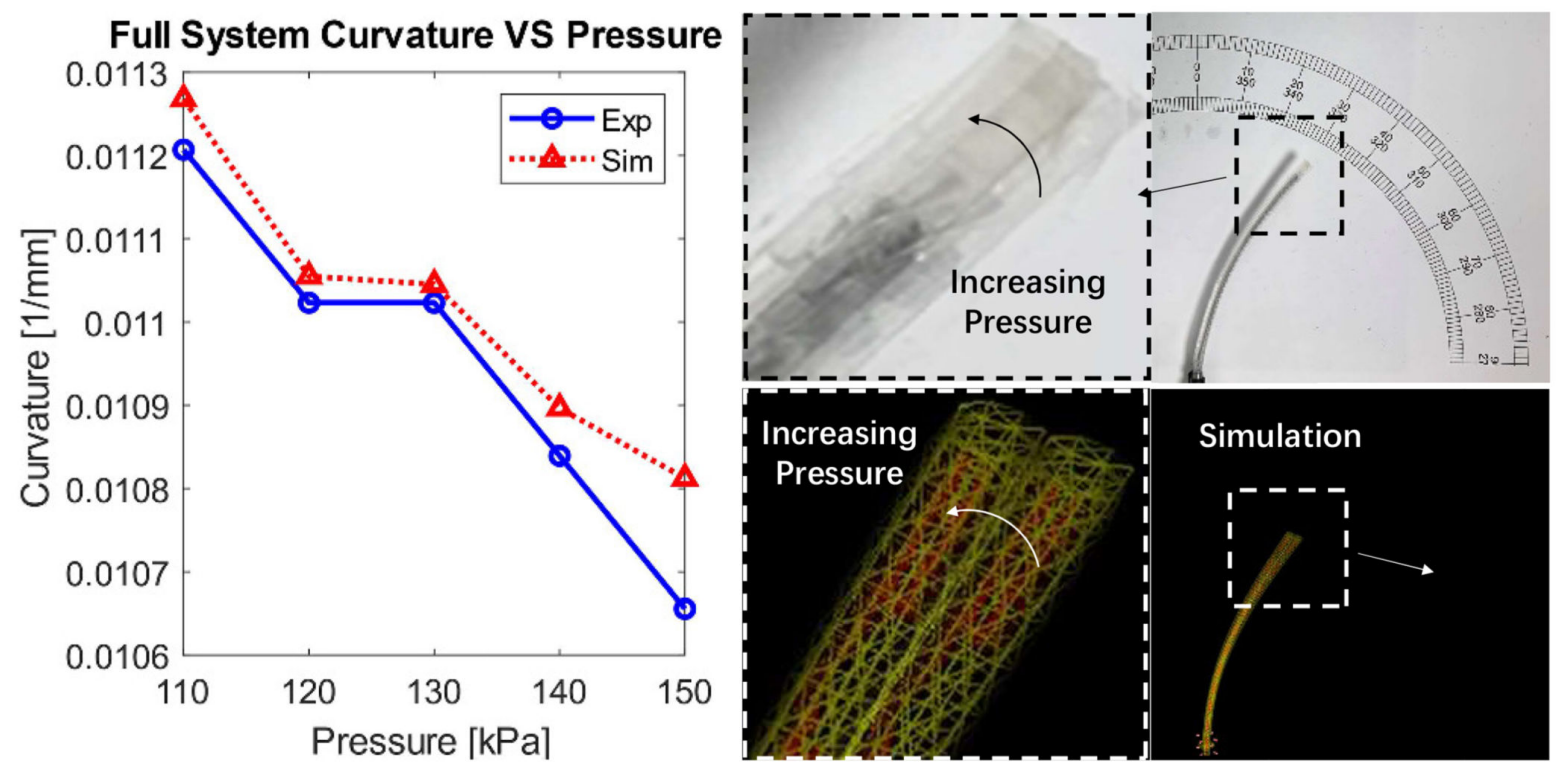
System curvature change vs. pressure with maximum tendon pulling.

The steerable catheter was deflected by pulling its tendon by 4. 9mm. Then, the growing sheath absolute pressure was regulated between 110 kPa to 150 kPa, with intervals of 10 kPa. The curvature change with varying pressure was recorded experimentally and in simulations. The results are shown in Fig. 10.

It is observed that the curvature of the growing robot decreased with increasing pressure both in the physical experiments and simulations. As expected, increasing the inner pressure of the sheath increased the overall stiffness of the growing robot, thus making its deflection harder and leading to reduced curvature. Overall, the slopes of the recorded quantities in experiments and simulations, calculated after a linear fit, are in good agreement. The MAE (%) between the experimental and simulation results is 0.066m^−1^ (0.61%).

The acceptable localisation error for a mammography task is reported to be about 5 mm [23]. Here, the MAE between the robot tip position in simulations and experiments was 1.16 mm that shows the adequate performance of the proposed modeling framework for a mammography task. However, the simulations for the robot growing length showed inadequate performance with an MAE of 11.89 mm.

#### 2) Consideration of Tip Load

The experiment was repeated introducing the effect of a tip force, both in the presence and absence of tendon tension. The magnitude of the forces considered ranged from 2 g to 5 g, and the orientation was either towards the catheter’s pattern side or opposite of it.

**Fig. 11:**
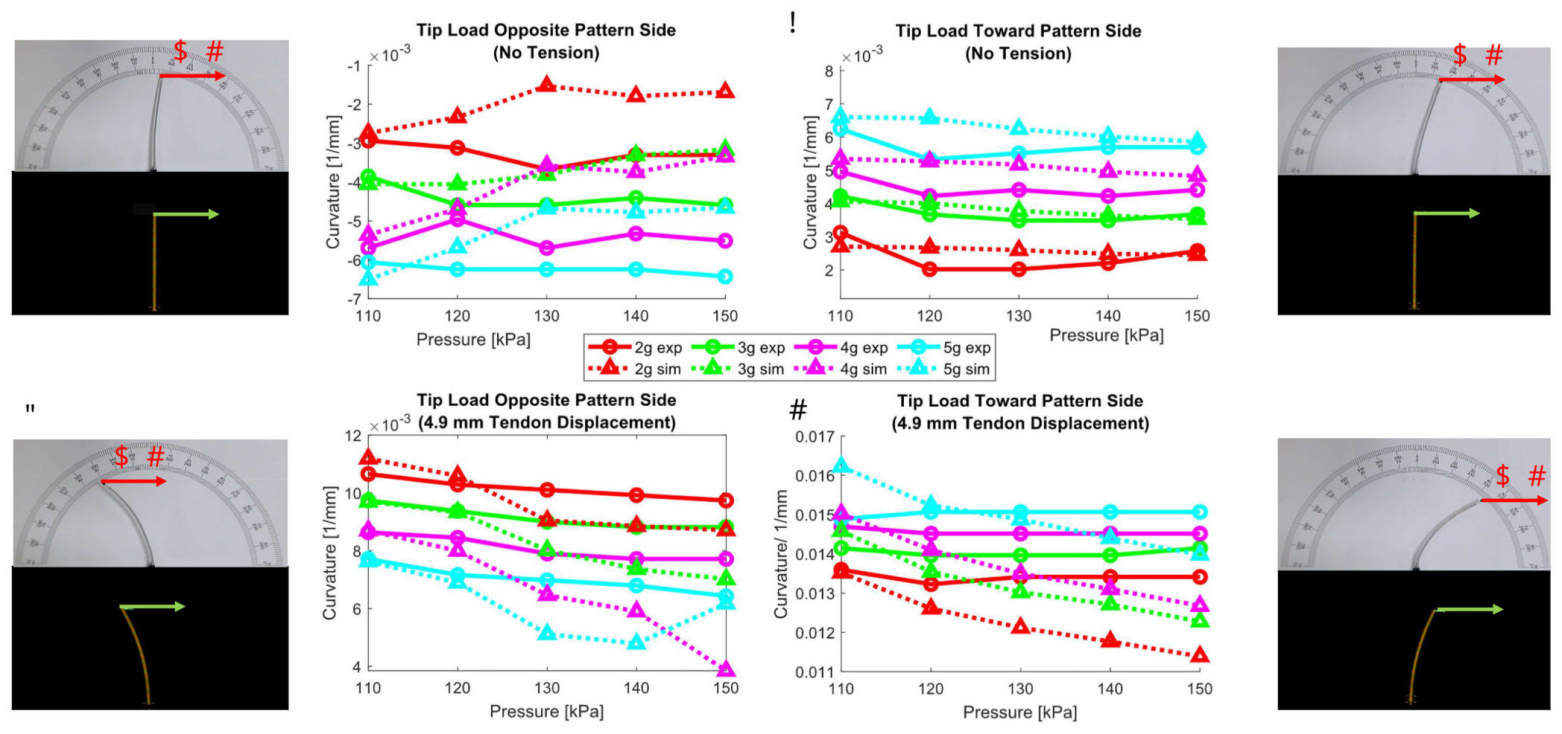
Initial curvature of the growing robot under loading that is (a) opposite to and (b) towards the deflection. Deflected curvature of the growing robot with tendon pulling of 4.9 mm under loading that is (c) opposite to and (d) towards the deflection.

Cases without tendon tension but with increasing pressures and loads are labelled as “initial” and are depicted in Fig. 11(a),(b) (Top row). Cases also including tendon tension are labelled as “deflected” and are depicted in Fig. 11(c),(d) (Bottom row). Results from experiments on the physical robot are plotted in solid lines while the results from the SOFA simulation are in dotted lines.

##### Absence of Tendon Tension

The results of Fig. 11(a),(b) show that for all applied forces and pressures, the SOFA simulation captures well the experiment with an MAE (%) of 1.12m^−1^ (24.20%) for the opposite loading case, and 0.46 m^-1^ (12.39%) for the toward loading case.

##### Presence of Tendon Tension

The results depicted in Fig. 11(c),(d) similarly show that when the full complexity of the system is considered, i.e. pressure induced growth, catheter, and bending, the SOFA simulation and experiment are once again in good agreement. Notably, the MAE (%) is 1.10m^-1^ (12.57%) for the opposite loading case, and 0.95 m^-1^ (6.73%) for the toward loading case.

The results are better when tendon tension and external load increase. This is expected, as in those cases the effects of manufacturing and alignment inaccuracies are reduced in favour of the increased bending. Overall, in conclusion, the fidelity of the developed model is on the order of 10-20% MAE, which is appropriate for a robot of this complexity.

Some contradicting trends were observed between the simulation and experimental results that highlight the complexity of physics-based modeling of a miniature growing robot. For example, despite the experimental results in Fig. 11(a), the increased internal pressure is expected to reduce the system curvature by straightening the structure as observed in the simulations. An asymmetric pre-tensions in the sheath structure might be a cause for such behavior.

### E Navigation Experiments

The final showcase entails the simulated navigation of MAMMOBOT within a system of vertically arranged pegs and a mammary duct replica. To ensure reliable navigation experiments in simulation extra functionalities, catheter base and sheath tip rotations, were added to the original scene. These allow the steerable catheter to be bent in any direction.

#### 1) Benchtop example

**Fig. 12:**
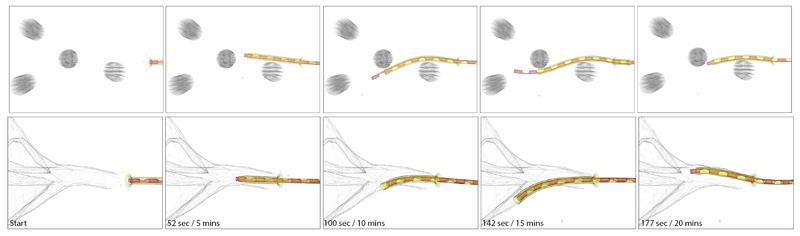
Deployment of the robot within simulated scenes. Captions indicate the time elapsed within the simulation, and the actual computation time. **Top row:** Rigid peg example. **Bottom row:** Mammary ducts example.

We are considering 4 rigid pegs of 5 mm diameter distributed within the scene. The robot can steer around them and interact with them to induce additional bending. A series of snapshots can be seen in Fig. 12 (Top row) while the accompanying video shows the full deployment. The system’s navigation also reflects the duty cycle approach and its limitations, reported in [5], manifesting in the protrusion of the catheter after significant sheath extension.

#### 2) Intraductal deployment

The ducts are a network-like structure with a small diameter of approximately 2mm to 3mm. The mammary duct network was reproduced from [5]. Remarkable locomotion and steering capabilities are required by its confined structure to access deep branches for diagnosis or treatment. A simulated deployment can be seen in Fig. 12 (Bottom row) and the accompanying video.

## V Discussion And Conclusions

The SOFA framework offers a powerful simulation environment to model physical interactions of eversion growing robots with their environments. Our simulation results using SOFA demonstrate comprehensive behavior of a miniature steerable soft growing robot during steering, growing, external loading and pressure stiffening, when compared with experiments.

We have identified limitations of a physics-based model for a miniature eversion growing robot that need to be addressed in the future. We could not capture the sheath growing physics based on FEM volumetric or planar meshes. Growing of the system could get unstable under large time steps. A large growing velocity could result in the sheath base folding over.

Finally, the chosen spring based contacts were limited to model internal friction via damping update during eversion. Nevertheless, dry friction represents a complex phenomenon with transition between different states. For this reason, we evaluated most of the contact models available in SOFA, such as friction constraint contact model, but without finding a computationally tractable solution. Our results highlights the need for a dedicated and optimized collision pipeline to model the internal friction of everting systems. Possible solutions would imply the use of Cosserat rod based bundles or graphic based contact solvers such as [24].

## Figures and Tables

**Table I:** Growing Robot Parameters In Simulation

	Catheter	Sheath	tendon
**Material**	Nitinol	LDPE	Nitinol
**Inner Dia.**	1.50 mm	2.95 mm	-
**Outer Dia.**	1.85 mm	3 mm	0.35 mm
**Full Length**	95 mm	95 mm	95 mm
**Thickness**	0.175 mm	35 um	-
**Total Mass**	0.215 g	0.056 g	0.059 g
**Young’s Modulus**	20 GPa	0.3 GPa	20 GPa
**Mesh Stiffness**	2e10* N/m	3e8* N/m	2e9* N/m
**Mesh Damping**	400* Ns/m	40* Ns/m	400* Ns/m

* identified values through experiments.

**Table II:** Error Analysis

Setup	Experiments	MAE (%)
System	Deflection via Varying Pressure	0.066 *m* ^−1^ (0.61%)
	Straight & Opposite Loading	1.12 *m* ^−1^ (24.20%)
	Straight & Toward Loading	0.46 *m* ^−1^ (12.39%)
	Deflected & Opposite Loading	1.10 *m* ^−1^ (12.57%)
	Deflected & Toward Loading	0.95 *m* ^−1^ (6.73%)
Catheter	Deflection via Tendon Pulling	0.55 *m* ^−1^ (20.78%)
	Deflection via Opposite Loading	0.72 *m* ^−1^ (27.51%)
	Deflection, Toward Loading	0.43 *m* ^−1^ (24.14%)
Sheath	Deflection via Gravity	6.39° (13.46%)
	Final Grown Length	11.89mm (17.97%)
	Average Growing Speed	0.46mm/s (51.03%)

## Footnotes

1
https://github.com/RViMLab/2022-RAL-SOFA-soft-growing-robots
